# Identification of new Wilms tumour predisposition genes: an exome sequencing study

**DOI:** 10.1016/S2352-4642(19)30018-5

**Published:** 2019-05

**Authors:** Shazia Mahamdallie, Shawn Yost, Emma Poyastro-Pearson, Esty Holt, Anna Zachariou, Sheila Seal, Anna Elliott, Matthew Clarke, Margaret Warren-Perry, Sandra Hanks, John Anderson, Simon Bomken, Trevor Cole, Roula Farah, Rhoikos Furtwaengler, Adam Glaser, Richard Grundy, James Hayden, Steve Lowis, Frédéric Millot, James Nicholson, Milind Ronghe, Jane Skeen, Denise Williams, Daniel Yeomanson, Elise Ruark, Nazneen Rahman

**Affiliations:** a Division of Genetics and Epidemiology, Institute of Cancer Research, London, UK; b Department of Haematology and Oncology, Great Ormond Street Hospital for Children NHS Foundation Trust, London, UK; c The Great North Children's Hospital, Newcastle upon Tyne Hospitals NHS Foundation Trust, Newcastle upon Tyne, UK; d Birmingham Women's and Children's NHS Foundation Trust, Birmingham, UK; e Department of Paediatrics, Saint George Hospital University Medical Centre, Beirut, Lebanon; f Department of Paediatric Hematology and Oncology, Saarland University Hospital, Homburg, Germany; g School of Medicine, University of Leeds, Leeds Institute of Data Analytics, Leeds, UK; h Children's Brain Tumour Research Centre, University of Nottingham, Queen's Medical Centre Nottingham, Nottingham, UK; i Department of Oncology, Alder Hey Children's NHS Foundation Trust, Liverpool, UK; j Department of Paediatric Oncology and Haematology, Bristol Royal Hospital for Children, Bristol, UK; k CIC 1402, Paediatric Oncology and Heamatology, Centre of Clinical Investigation, Poitiers, France; l Paediatric Oncology and Haematology, Addenbrooke's Hospital, Cambridge Biomedical Campus, Cambridge, UK; m Department of Paediatric Oncology, Royal Hospital for Children, Queen Elizabeth University Hospital, Glasgow, UK; n Starship Children's Hospital, Auckland, New Zealand; o Department of Haematology and Oncology, Sheffield Children's Hospital, Sheffield, UK; p Cancer Genetics Unit, Royal Marsden NHS Foundation Trust, London, UK

## Abstract

**Background:**

Wilms tumour is the most common childhood renal cancer and is genetically heterogeneous. While several Wilms tumour predisposition genes have been identified, there is strong evidence that further predisposition genes are likely to exist. Our study aim was to identify new predisposition genes for Wilms tumour.

**Methods:**

In this exome sequencing study, we analysed lymphocyte DNA from 890 individuals with Wilms tumour, including 91 affected individuals from 49 familial Wilms tumour pedigrees. We used the protein-truncating variant prioritisation method to prioritise potential disease-associated genes for further assessment. We evaluated new predisposition genes in exome sequencing data that we generated in 334 individuals with 27 other childhood cancers and in exome data from The Cancer Genome Atlas obtained from 7632 individuals with 28 adult cancers.

**Findings:**

We identified constitutional cancer-predisposing mutations in 33 individuals with childhood cancer. The three identified genes with the strongest signal in the protein-truncating variant prioritisation analyses were *TRIM28, FBXW7*, and *NYNRIN*. 21 of 33 individuals had a mutation in *TRIM28*; there was a strong parent-of-origin effect, with all ten inherited mutations being maternally transmitted (p=0·00098). We also found a strong association with the rare epithelial subtype of Wilms tumour, with 14 of 16 tumours being epithelial or epithelial predominant. There were no *TRIM28* mutations in individuals with other childhood or adult cancers. We identified truncating *FBXW7* mutations in four individuals with Wilms tumour and a de-novo non-synonymous *FBXW7* mutation in a child with a rhabdoid tumour. Biallelic truncating mutations in *NYNRIN* were identified in three individuals with Wilms tumour, which is highly unlikely to have occurred by chance (p<0·0001). Finally, we identified two de-novo *KDM3B* mutations, supporting the role of *KDM3B* as a childhood cancer predisposition gene.

**Interpretation:**

The four new Wilms tumour predisposition genes identified—*TRIM28, FBXW7, NYNRIN*, and *KDM3B*—are involved in diverse biological processes and, together with the other 17 known Wilms tumour predisposition genes, account for about 10% of Wilms tumour cases. The overlap between these 21 constitutionally mutated predisposition genes and 20 genes somatically mutated in Wilms tumour is limited, consisting of only four genes. We recommend that all individuals with Wilms tumour should be offered genetic testing and particularly, those with epithelial Wilms tumour should be offered *TRIM28* genetic testing. Only a third of the familial Wilms tumour clusters we analysed were attributable to known genes, indicating that further Wilms tumour predisposition factors await discovery.

**Funding:**

Wellcome Trust.

## Introduction

Wilms tumour is a childhood kidney tumour that affects one in 10 000 children. Its histology is similar to that of the developing kidney and is typically triphasic, with blastemal, stromal, and epithelial components.^1^ Biphasic tumours, with two of the three components, and monomorphic tumours consisting of only one component also occur but are rarer.^1^

Wilms tumour is primarily a non-familial condition, with only about 2% of affected individuals having a relative with the tumour.^2^ Given its rarity, inherited causes—rather than chance—are assumed to underlie familial clusters, and several have been reported, including constitutional mutations in the genes *WT1, CTR9*, and *REST*.^2^, ^3^, ^4^ Wilms tumour is also known to be associated with many genetic conditions, including the WAGR, Denys-Drash, Beckwith-Wiedemann, Simpson-Golabi-Behmel, Perlman, mosaic variegated aneuploidy, hereditary hyperparathyroidism-jaw tumour, Li-Fraumeni, DICER1, and Bohring-Opitz syndromes, Fanconi anaemia, and *PIK3CA*-related overgrowth spectrum.^2^, ^3^, ^4^, ^5^, ^6^, ^7^, ^8^, ^9^, ^10^, ^11^ These conditions are diverse in their clinical and histological associations, inheritance patterns, and mutational mechanisms of pathogenicity. The underlying predisposition genes have many different functions and are involved in diverse biological processes. The identification of these genes and investigations into their role in Wilms tumour predisposition have led to fundamental insights into developmental, cellular, and oncological mechanisms and have important clinical implications for individuals with Wilms tumour and their families.^2^, ^3^, ^4^, ^5^, ^6^, ^7^, ^8^, ^9^, ^10^, ^11^ Furthermore, many causes of familial and syndromic Wilms tumour also contribute to non-familial, non-syndromic Wilms tumour.^2^, ^4^, ^5^

**Research in context**

**Evidence before this study**

Wilms tumour is a rare childhood kidney tumour. We searched PubMed for papers in English with the terms “Wilms” AND “genetic” OR “mutation” OR “familial” OR “syndrome”, yielding 2801 papers that we reviewed to identify those relevant to genetic predisposition to Wilms tumour. This review identified 17 genes previously shown to predispose to Wilms tumour and showed that further Wilms tumour predisposition genes must exist, because many syndromic cases and familial clusters have not been explained.

**Added value of this study**

To our knowledge, this study is the largest exome sequencing study to date of individuals with Wilms tumour, involving 890 individuals, including 91 individuals from 49 familial Wilms tumour pedigrees. We identified four new Wilms tumour predisposition genes, *TRIM28, FBXW7, KDM3B*, and *NYNRIN*. We showed that *FBXW7* and *KDM3B* are pleiotropic cancer predisposition genes, and that *KDM3B* and *NYNRIN* might also cause non-malignant phenotypes, particularly intellectual disability. Our study identified *TRIM28* as a major Wilms tumour predisposition gene, making a similar contribution to familial and unselected Wilms tumour as those of constitutional *WT1* and *REST* mutations. We also found an association between *TRIM28* mutations and epithelial histology and a strong parent-of-origin-effect, because all inherited *TRIM28* mutations were maternally transmitted. Functional enrichment analyses revealed remarkable diversity in the biological pathways affected by Wilms tumour predisposition genes. We also found limited overlap between the 21 constitutionally mutated Wilms tumour predisposition genes and 20 genes somatically mutated in Wilms tumour.

**Implications of all the available evidence**

This study provides new insights into the causes of Wilms tumour and describes the overall landscape of Wilms tumour predisposition. Wilms tumour shows remarkable genetic heterogeneity and aetiological complexity, which have substantial clinical impact. Genetic testing should be made available to individuals with Wilms tumour, but will need to encompass both broad genetic testing, for example by exome sequencing, and testing for 11p15 epigenetic abnormalities. Moreover, our findings suggest that more Wilms tumour predisposition genes are likely to exist, which will have relevance for future research and clinical testing.

Research over the past 25 years has led to tremendous advances in our knowledge of Wilms tumour predisposition. However, available evidence suggests that our knowledge is still incomplete and that further predisposition factors remain to be discovered. In particular, the cause of many familial clusters is still unknown.^4^ In this study, we aimed to use exome sequencing to identify new Wilms tumour predisposition genes and to characterise and contextualise the genetic landscape of such predisposition.

## Methods

### Study design and participants

**Figure 1:**
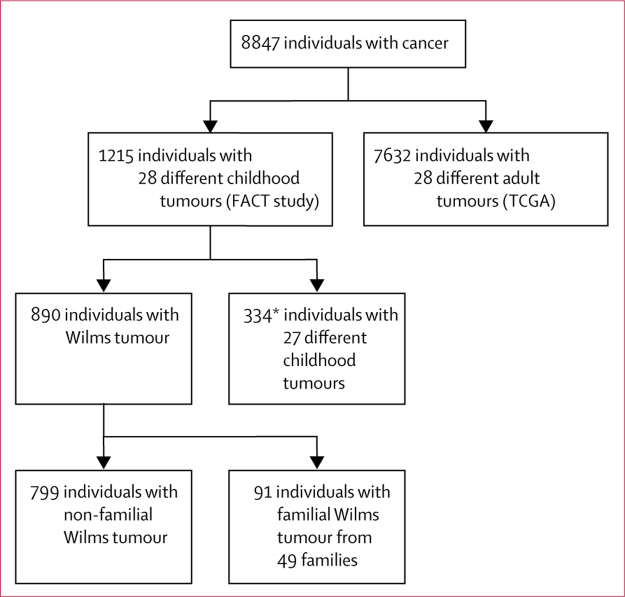
Cancer cohorts investigated Wilms tumour families are pedigrees in which two or more individuals had Wilms tumour. FACT=Factors Associated with Childhood Tumours. TCGA=The Cancer Genome Atlas. *Nine individuals had two different childhood tumours.

In this exome sequencing study, we included participants recruited to the Factors Associated with Childhood Tumours (FACT) Study. All children with a childhood solid tumour from the UK were eligible for participation in the FACT study; children with familial childhood cancer anywhere in the world were also eligible for participation in the FACT study. We analysed lymphocyte DNA from 1215 individuals with 28 different childhood tumours, of whom 1206 had one childhood tumour and nine individuals had two different childhood tumours (appendix). This cohort included 890 individuals with Wilms tumour: 799 had non-familial disease and 91 were from 49 familial Wilms tumour pedigrees in which two or more individuals had Wilms tumour due to an unknown genetic or epigenetic cause (figure 1; appendix). Most participants with Wilms tumour were from the UK and, therefore, were likely to have been treated with chemotherapy before surgery. We used constitutional (germline) exome data from 7632 individuals with 28 different adult cancers available from The Cancer Genome Atlas (TCGA) on May 13, 2014 (figure 1; see appendix for the types and number of adult cancers interrogated). As reference data, we used the Exome Aggregation Consortium (ExAC) data, version 3, accessed on Nov 13, 2015 (excluding the TCGA samples),^12^ and the ICR1000 UK exome series.^13^ We generated and analysed the ICR1000 UK exome series and childhood cancer sample data from the FACT participants by use of consistent sequencing and analytical processes.

The FACT study was approved by the London Multicentre Ethics Committee (05/MRE02/17), and its collaborators are listed in the appendix. Written informed consent was obtained from all participants, their parents or guardians, or both, as appropriate (age cutoff for consent was 18 years).

### Procedures

We did exome sequencing in samples from all childhood cancer probands and 119 individuals from 49 familial Wilms tumour pedigrees who did not have Wilms tumour, by using 50 ng genomic DNA and the Nextera DNA sample preparation kit (Illumina, San Diego, CA, USA) or 1·5 μg genomic DNA and the TruSeq exome enrichment kit (Illumina). The captured libraries were amplified by PCR with the supplied paired-end PCR primers. Sequencing was done with HiSeq 2000 (Illumina) or HiSeq 2500 (Illumina). We used the OpEx v1.0 pipeline to do variant calling in childhood cancer, adult cancer (TGCA), and ICR1000 exome data.^14^ We also reannotated the variants in the ExAC data with the CAVA tool in OpEx, to ensure variant calling consistency across the different cohorts.^14^ We used the protein-truncating variant prioritisation method to prioritise potential disease-associated genes for follow-up; this is a proven strategy for identifying tumour suppressor genes in outbred populations, which we have used to identify several other cancer predisposition genes.^3^, ^4^, ^10^ We validated variants in *TRIM28, FBXW7, NYNRIN*, and *KDM3B* genes by use of Sanger sequencing in the probands and any available relatives, designing primers with BatchPrimer3. We used the QIAGEN Multiplex PCR kit (QIAGEN, Hilden, Germany) to prepare PCRs, and the resulting amplicons were bidirectionally sequenced with BigDye Terminator cycle sequencing kits (Thermo Fisher Scientific, Waltham, MA, USA) and an ABI 3730 sequencer (Life Technologies, Carlsbad, CA, USA). We analysed sequencing traces with Mutation Surveyor software and by visual inspection with Chromas, version 2.13. We validated the *CDC73* mutation with the TruSight Cancer panel (Illumina). We did in-silico analyses of variant pathogenicity with Alamut Visual, version 2.9.0.

### Statistical analysis

We used the methods described by Akawi and colleagues^15^ to obtain the probability of a family in our study having two protein-truncating variants in a given gene. The method uses the frequency of rare protein-truncating variants (allele frequency <0·001) in ExAC and the number of observed protein-truncating variants in a given gene to estimate the probability of an individual having two of these variants in that gene. The baseline prevalence of having two protein-truncating variants per gene is calculated as the proportion of rare protein-truncating variants squared. We observed two individuals with two protein-truncating variants and nine individuals with a single protein-truncating variant in *NYNRIN* among 844 individuals with Wilms tumour from the 890 included in this study. We used the R function to calculate the probability of observing two individuals with two *NYNRIN* protein-truncating variants: analyse_inherited_enrichment from the R package recessiveStats with hgnc=”NYNRIN”, chrom=“14”, counts$biallelic_lof=2, counts$lof_func=9, and cohort_n=844.

We used a binomial test—dbinom function in R—to calculate the probability of all ten *TRIM28* mutations with known inheritance being maternally inherited, assuming the baseline probability of inheriting the variant from either parent was 0·5.

We did a functional enrichment analysis with use of g:Profiler (version r1665_e85_eg32).^16^ We used the 21 predisposition genes described for Wilms tumour as our query set. We looked for enrichment among Gene Ontology molecular function terms and pathway gene sets from the Kyoto Encyclopedia of Genes and Genomes, requiring the size of the functional category to have a minimum of five genes and using the Benjamini-Hochberg correction for multiple testing p value as the significance threshold. The false discovery rate q values presented in this study are the Benjamini-Hochberg critical values.

For the somatic cancer driver comparisons, we used 20 genes reported to be somatically mutated in Wilms tumour. These included 17 established genes reported in more than one publication and three newly reported genes (*ACTB, BCOR, NONO*) with at least three somatic mutations in the TARGET discovery series.^17^ We used the COSMIC cancer gene census to establish which of the 21 Wilms tumour predisposition genes, and which of the 20 somatically mutated Wilms tumour driver genes, were also somatically mutated in other cancer types.

### Role of the funding source

The funder of the study had no role in study design, data collection, data analysis, data interpretation, or writing of the report. SM, SY, EH, and NR had full access to all the data. The corresponding author had full access to all the data in the study and had final responsibility for the decision to submit for publication.

## Results

We used the protein-truncating variant prioritisation method with dominant and recessive inheritance models to identify genes with different protein-truncating variants in 890 individuals with Wilms tumour (figure 1). The three genes with the strongest signal in the prioritisation analyses were *TRIM28, FBXW7*, and *NYNRIN*. We did Sanger sequencing to validate protein-truncating variants and rare non-synonymous variants of these genes in probands and any available samples from relatives to further evaluate their status as bona fide Wilms tumour predisposition genes.

**Figure 2:**
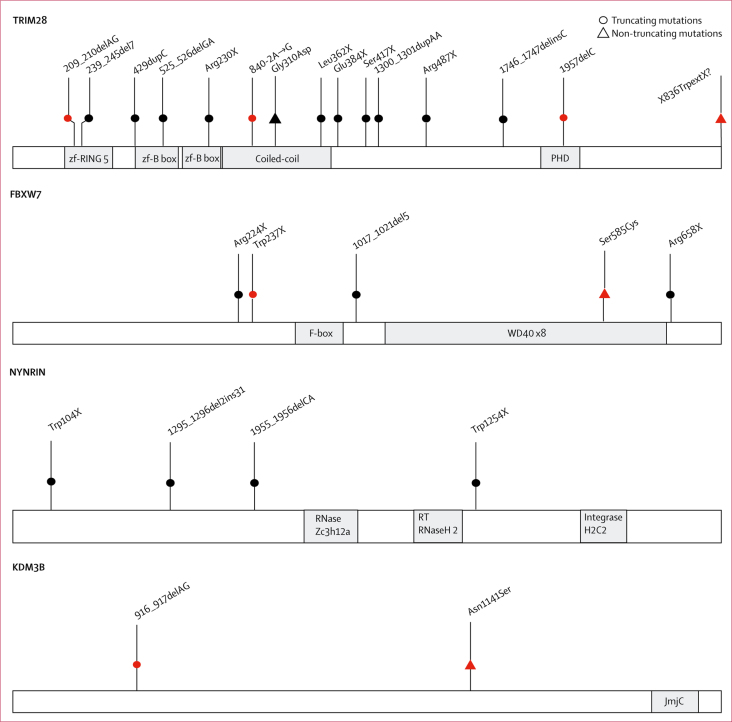
Schematic representations of *TRIM28, FBXW7, NYNRIN*, and *KDM3B* Schematic representations of encoded proteins are shown, with functional domains in grey. The position of cancer-predisposing mutations is shown above the protein. Red symbols denote de novo mutations.

We identified pathogenic truncating mutations of *TRIM28* in 17 individuals with Wilms tumour from 13 families (figure 2, table, appendix). At least three of these truncating mutations had arisen de novo. Protein-truncating variants in *TRIM28* are extremely rare in the general population, because the gene is highly intolerant to truncating variation, with a pLI score of 1·0 (pLI >0·9 indicates extreme intolerance to protein-truncating variants).^12^ We found no other cancers in individuals carrying *TRIM28* mutations. We also did not find any *TRIM28* protein-truncating variants in 334 individuals with 27 other childhood cancers or in 7632 individuals with adult cancers, suggesting that *TRIM28* pathogenic mutations primarily predispose to Wilms tumour. Of note, the *TRIM28* mutations in two families in our study (ID_0498 and ID_0506) were independently, and coincidentally, reported while we were preparing this manuscript.^18^ We identified a de-novo stop-loss mutation in another family (ID_7574), which we assumed to be pathogenic. Finally, in family ID_0477, which included six cases of Wilms tumour, we identified *TRIM28* 929G→A, leading to the protein change Gly310Asp (figure 2, table, appendix). We believe this mutation to be pathogenic because it is absent from public and in-house datasets, it segregates with Wilms tumour in the family, it is predicted to be deleterious by in silico tools, and it is at a crucial residue within the coiled-coil domain of TRIM28 that is reported to interact with AMER1, which is encoded by a gene somatically mutated in Wilms tumour.^19^

**Table:** Molecular and clinical features of individuals with mutations in *TRIM28, FBXW7, NYNRIN, KDM3B*, or *CDC73*

	**Sex**	**Mutations (protein change)**	**Inheritance**	**Tumour type, age at diagnosis (months)**	**Unilateral or bilateral**	**Histology**	**Other**	**Status, age (years)**
**TRIM28**								
ID_0477_01	F	929G→A (Gly310Asp)	Maternal	WT, 24	Unilateral	Epithelial predominant	..	..
ID_0477_02	M	929G→A (Gly310Asp)	Maternal	WT, 84	Unilateral	Epithelial	..	..
ID_0477_03	F	929G→A (Gly310Asp)	Maternal	WT, 93	Unilateral	NA	..	..
ID_0498_01	M	1746_1747delinsC	Maternal	WT, 8	Unilateral	Epithelial	..	Alive, 30
ID_0498_02	F	1746_1747delinsC	Maternal	WT, 5	Unilateral	Epithelial	..	Alive, 29
ID_0498_03	F	1746_1747delinsC	NA	WT, 6	Unilateral	Epithelial	..	..
ID_0487_01	M	429dupC	Maternal	WT, 15	Unilateral	Epithelial predominant	..	Alive, 19
ID_0487_02	M	429dupC	NA	WT, 18	Unilateral	NA	..	..
ID_0506_01	M	525_526delGA	Maternal	WT, 39	Unilateral	Epithelial	..	Alive, 23
ID_0506_02	F	525_526delGA	Maternal	WT, 8	Bilateral	Epithelial	..	Alive, 20
ID_7487_01	F	239_245del7	Maternal	WT, 118	Unilateral	Epithelial predominant, diffuse anaplasia	..	Died, 12
ID_1982	M	1957delC	De novo	WT, 11	Bilateral	Epithelial predominant	..	Alive, 15
ID_6530	M	209_210delAG	De novo	WT, 15	Unilateral	Epithelial and blastemal	Autism, speech delay	Alive, 6
ID_1969	M	840–2A→G	De novo	WT, 118	Unilateral	Epithelial and blastemal	..	Alive, 19
ID_7574	M	2508A→G (X836TrpextX?)*	De novo	WT, 13	Unilateral	Epithelial predominant	Autism, intellectual disability	..
ID_0902	F	1250C→A (Ser417X)	Maternal	WT, 12	Unilateral	Epithelial predominant	..	..
ID_0692	F	1459C→T (Arg487X)	NA	WT, 13	Bilateral	NA	..	Alive, 36
ID_6671	F	688C→T (Arg230X)	NA	WT, 10	Bilateral	Epithelial predominant	Chronic kidney disease	Alive, 6
ID_0796	F	1085T→A (Leu362X)	NA	WT, 61	Unilateral	NA	..	Alive, 33
ID_0866	F	1300_1301dupAA	NA	WT, 90	Unilateral	Epithelial predominant	..	Alive, 29
ID_0936	M	1150G→T (Glu384X)	NA	WT, 8	Unilateral	NA	..	..
**FBXW7**								
ID_0811	M	710G→A (Trp237X)	De novo	WT, 76	Unilateral	NA	Osteosarcoma at 39 years	Died, 39
ID_2084_01	M	1972C→T (Arg658X)	NA	WT, 42	Unilateral	Focal anaplasia	Relapse at 66 months	..
ID_0592	F	1017_1021del5	Paternal	WT, 28	Unilateral	NA	hypotonia	Alive, 18
ID_1227	F	670C→T (Arg224X)	NA	WT, 73	..	NA	..	Died, 7
ID_7520	M	1753A→T (Ser585Cys)	De novo	Rhabdoid, 40	..	Extra-renal rhabdoid with *INI1* loss	Two febrile convulsions	Alive, 5
**NYNRIN**								
ID_0493_01	M	1955_1956delCA	Paternal	WT, 24	Unilateral	Blastemal predominant	Inguinal hernia	..
ID_0493_01	..	3761G→A (Trp1254X)	Maternal	..	..	..	..	..
ID_0493_02	M	1955_1956delCA	Paternal	WT, 24	Unilateral	Triphasic	..	..
ID_0493_02	..	3761G→A (Trp1254X)	Maternal	..	..	..	..	..
ID_6049	M	311G→A (Trp104X)	Maternal	WT, 34	Unilateral	Triphasic	Epilepsy, hypothyroidism, intellectual disability	Alive, 11
ID_6049	..	1295_1296del2ins31	Paternal	..	..	..	..	..
**KDM3B**								
ID_7225	F	3422A→G (Asn1141Ser)	De novo	WT, 49	Bilateral	NA	Hyperpigmentation	..
ID_2086	M	916_917delAG	De novo	Hepatoblastoma, 131	..	NA	Autism, abnormal pigmentation, intellectual disability	..
**CDC73**								
ID_6491_01	F	878dupA	Paternal	WT, 192	Unilateral	Epithelial predominant	Convergent strabismus	Alive, 21
ID_6491_02	M	878dupA	NA	WT, 96	Unilateral	NA	..	Alive, 48

Pedigrees and chromatograms are shown in the appendix. F=female. WT=Wilms tumour. M=male. NA=not available.

* The stop codon (X) at position 836 is changed to Trp, extending the protein by an unknown number of amino acids (?).

We established that ten of the *TRIM28* mutations had been inherited, and that in all cases, the mutation had been transmitted from the mother, a significant association (p=0·00098). Pathology information was available for 16 tumours, of which 14 were epithelial or epithelial-predominant (table).

We identified truncating *FBXW7* mutations in four individuals with Wilms tumour, of which one was de novo (in ID_0811), one had been inherited from an unaffected father (in ID_0592), and two were of unknown provenance (in ID_2084 and ID_1227; figure 2, table, appendix). *FBXW7* is highly intolerant to protein-truncating variants (pLI=1·00) and these data suggest that *FBXW7* is a Wilms tumour predisposition gene. Two of the four individuals with truncating *FBXW7* mutations have died (table). Additionally, ID_2084 was treated for Wilms tumour at 3·5 years of age, but relapsed when he was 5·5 years old. We did not find truncating *FBXW7* mutations in individuals with other childhood or adult cancers. However, we identified a de novo non-synonymous mutation, 1753A→T (protein change Ser585Cys), in a child with an extra-renal rhabdoid tumour (ID_7520), which we assumed to be pathogenic.

We identified biallelic truncating mutations in *NYNRIN* in three children from two families (ID_0493 and ID_6049; figure 2, table, appendix). Each parent was heterozygous for one of the mutations. These mutations were absent from ExAC and the ICR1000 series. We found no individuals with two *NYNRIN* truncating mutations in the ICR1000 series and no homozygous protein-truncating variants in ExAC (individual-level data is not available for ExAC, therefore it is not possible to know if anyone had two different protein-truncating variants). Additionally, the probability of finding two different families with the same phenotype and two truncating *NYNRIN* mutations by chance is 4·0 × 10^−9^. One of the affected children had an inguinal hernia and another had epilepsy, hypothyroidism, and intellectual disability (table). It is unclear whether any of these additional clinical features are related to the biallelic *NYNRIN* mutations. We did not identify biallelic *NYNRIN* protein-truncating variants in individuals with other childhood or adult cancers.

In addition to the agnostic protein-truncating variant prioritisation analyses, we reviewed the exome data in genes proposed as possible childhood cancer predisposition genes, identified through a systematic review of 19 171 genes for links to Mendelian disease. This review led us to the identification of two de-novo *KDM3B* mutations, a non-synonymous mutation in a child with Wilms tumour and a hyperpigmented lesion on her buttock (ID_7225) and a truncating mutation in a child with hepatoblastoma, hyperpigmentation and hypopigmentation, autism, and intellectual disability (ID_2086; figure 2, table, appendix). In 2018, Diets and colleagues^20^ reported a *KDM3B* truncating mutation in a girl with acute myeloid leukaemia, mild intellectual disability, and hip dysplasia and a de novo non-synonymous *KDM3B* mutation in a boy with Hodgkins lymphoma and moderate intellectual disability. *KDM3B* is highly intolerant to both protein-truncating variants (pLI=1·00) and non-synonymous variation (Z=4·99; the Z score is the deviation of observation from expectation for non-synonymous variants). Taken together, these data provide strong evidence that *KDM3B* is a childhood cancer predisposition gene.

**Figure 3:**
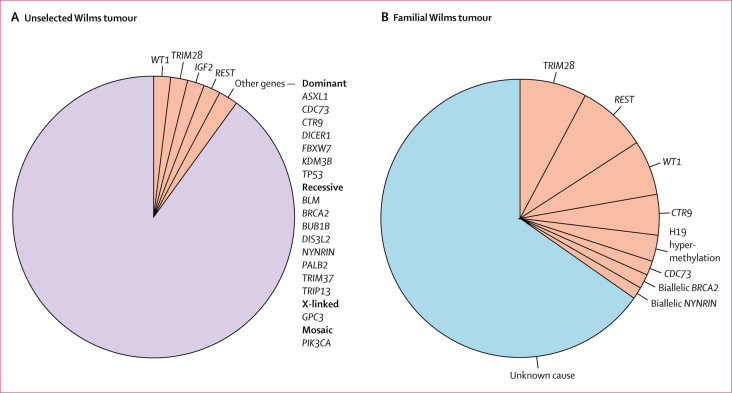
Contribution of constitutional mutations to unselected and familial Wilms tumour (A) About 10% of unselected Wilms tumours are due to constitutional mutations in one of 21 genes (pink). (B) A third of familial Wilms tumours are explicable by known Wilms tumour predisposition factors (pink) and two thirds are of unknown cause (blue).

These new discoveries bring the number of constitutionally mutated genes confirmed as Wilms tumour predisposition genes to 21. We estimate that, together, these constitutional events contribute to about 10% of unselected Wilms tumour (figure 3). Four contributors—*WT1, TRIM28, REST*, and 11p15 epimutations and uniparental disomy that result in biallelic *IGF2* expression—each account for about 2%.^2^, ^4^, ^5^ The remaining 17 are very rare and, together, probably account for no more than 2% of unselected Wilms tumours.^2^, ^3^, ^4^, ^6^, ^7^, ^8^, ^9^, ^10^, ^11^ Functional enrichment analysis highlighted nucleic acid metabolism, chromosome organisation, chromatin or histone modification, and negative regulation of cellular processes as important pathways underlying Wilms tumour predisposition (appendix).

We have investigated 65 families with two or more cases of Wilms tumour over the last 20 years, including the 49 familial Wilms tumour pedigrees in this study (appendix). In two families, we found a constitutional predisposing mutation in one individual with Wilms tumour, but not their affected relative. We have identified causative constitutional mutations in 22 (35%) of the remaining 63 families (figure 3). The most common of which were mutations in *REST* (five [8%] of 63), *TRIM28* (five [8%] of 63), and *WT1* (four [6%] of 63). *CTR9* mutations were present in three families and H19 hypermethylation was found in two families. Biallelic *BRCA2* mutations, biallelic *NYNRIN* mutations, and a *CDC73* mutation were found in one family each. We identified the *CDC73* mutation through this present study (table, appendix). *CDC73* is an established Wilms tumour predisposition gene but, to our knowledge, has not previously been associated with familial Wilms tumour. We did not find any cause in two thirds of the families (41 [65%] of 63).

**Figure 4:**
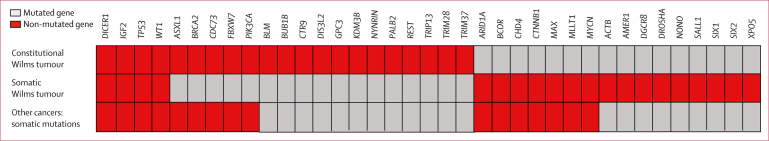
Overlap of constitutionally and somatically mutated Wilms tumour genes

Finally, we assessed the overlap between the 21 Wilms tumour predisposition genes and 20 somatically mutated Wilms tumour driver genes (figure 4). Only four genes—*WT1, IGF2, TP53*, and *DICER1*—promoted Wilms tumour oncogenesis in both contexts, and all four were also somatically altered in other cancers. A further five constitutionally mutated genes—*PIK3CA, FBXW7, ASXL1, BRCA2*, and *CDC73*—were somatically mutated in other cancer types but have not been proven to be somatic drivers in Wilms tumour. The remaining 12 Wilms tumour predisposition genes are not known to be somatically mutated cancer drivers.

## Discussion

To our knowledge, this is the largest exome sequencing study of individuals with Wilms tumour to date, including 890 affected individuals. Our analyses found three autosomal dominant Wilms tumour predisposition genes—*TRIM28, FBXW7*, and *KDM3B*—and one autosomal recessive Wilms tumour predisposition gene, *NYNRIN*. Constitutional *TRIM28* mutations join constitutional *WT1* and *REST* mutations as a relatively common contributor to Wilms tumour predisposition, accounting for about 8% of familial Wilms tumour and about 2% of unselected Wilms tumour.^2^, ^4^ We found a strong association between *TRIM28* mutations and epithelial Wilms tumour, with most individuals with a *TRIM28* mutation having Wilms tumour of predominantly epithelial histology. This suggests that *TRIM28* mutations make a sizeable contribution to epithelial Wilms tumour, and we recommend that all children with this rare favourable subtype of Wilms tumour should be offered *TRIM28* gene testing.

Familial Wilms tumour pedigrees due to *TRIM28* mutations showed incomplete penetrance and a strong parent-of-origin effect, because all inherited mutations were maternally transmitted. *TRIM28* is not imprinted, but it is located close to *PEG3,* which is imprinted and paternally expressed.^21^ At the organism level, *PEG3* promotes growth but at the cellular level, it is a putative tumour suppressor.^21^ A possible explanation for the maternal bias of *TRIM28* mutations is that somatic inactivation of the paternal wild-type *TRIM28* allele, through mitotic recombination, also leads to loss of the paternally expressed *PEG3*, thus promoting tumourigenesis. There is precedence for this model in cancer predisposition: *SDHD* mutations predispose to phaeochromocytoma almost exclusively when inherited paternally.^22^ The combination of somatic loss of the maternal wild-type *SDHD* allele and maternally expressed growth inhibitory genes at the *IGF1–H19* imprinting region has been proposed as the explanation for this pattern.^22^ However, a factor against this model for *TRIM28* is that the loss of heterozygosity in the tumour from ID_506_01 did not appear to include *PEG3*.^18^ Given the prevalence of *TRIM28* mutations in Wilms tumour, confirmation of the parent-of-origin bias we observed should be possible in the near future. If this parent-of-origin effect is supported, DNA methylation analyses at the *PEG3* imprinting control region and loss of heterozygosity and *PEG3* expression analyses in tumours from individuals with *TRIM28* mutations might help to provide a mechanistic explanation. From a clinical perspective, establishing if the penetrance of *TRIM28* mutations is influenced by the parent-of-origin of the mutation is important, because this would have considerable impact on cancer risks and genetic counselling.

We provided evidence that constitutional *FBXW7* mutations predisposed to Wilms tumour and to other malignancies. Two of the five individuals carrying *FBXW7* mutations had a malignancy other than Wilms tumour. ID_0811 developed osteosarcoma as an adult, after having Wilms tumour. ID_7520 had a rhabdoid tumour and did not have Wilms tumour. The assessment of additional individuals with rhabdoid tumour and de-novo *FBXW7* mutations would be useful to further support the role of *FBXW7* in rhabdoid tumour predisposition. Furthermore, a woman with Hodgkin lymphoma, adult Wilms tumour, early-onset breast cancer, and a constitutional *FBXW7* deletion was reported in 2015,^23^ and a man with renal cell cancer and a constitutional t(3;4)(q21;q31) translocation disrupting *FBXW7* was reported in 2009.^24^ These data suggest that individuals with *FBXW7* mutations might be at risk of multiple childhood and adult cancers and will require ongoing close monitoring. Notably, we believe that the in-frame *FBXW7* variant reported^25^ in an individual with Wilms tumour is not pathogenic because it is not rare and the child also had a pathogenic *WT1* mutation.

*KDM3B* also appears to be a pleiotropic cancer predisposition gene. The four *KDM3B* pathogenic mutations reported to date have been associated with four different cancers: Wilms tumour and hepatoblastoma in our study, and acute myeloid leukaemia and Hodgkin lymphoma in the study by Diets and colleagues.^20^ Large-scale, broad mutation testing of *FBXW7* and *KDM3B* in individuals with cancer will probably be required to establish the full spectrum of associated cancers, because of the rarity of truncating variants and the challenges in interpreting non-synonymous variation in these genes. There are indications that *KDM3B* and *NYNRIN* mutations might cause non-malignant phenotypes, particularly intellectual disability. More data on the contribution of these genes to non-malignant conditions will probably become available over the next decade through extensive exome and genome sequencing being done in children with developmental disorders.

The four genes we reported here have different functions, and it is unclear why or how they predispose to Wilms tumour. *TRIM28* encodes a multidomain protein involved in the regulation of many cellular processes, including transcriptional repression, p53 degradation, pluripotency maintenance, autophagosome formation, epithelial-mesenchyme transition, and the DNA damage response.^26^ *TRIM28* is highly expressed in many cancers, and its inactivation has not been previously associated with oncogenesis. This might explain why inactivating *TRIM28* mutations seem to predispose to Wilms tumour alone. The mechanisms underlying this Wilms tumour predisposition are not known, but it is notable that TRIM28 is a major binding partner of AMER1, which is encoded by a gene that is frequently somatically mutated in Wilms tumour.^19^

*FBXW7* encodes the substrate recognition component of the E3-ubiquitin ligase SCF complex, which is responsible for recognising and binding phosphorylated substrates and regulating their turnover through proteosome degradation.^27^ *FBXW7* is an established tumour suppressor gene and frequently mutated in many cancers, particularly endometrial and gastrointestinal cancers.^27^ *FBXW7* is not a confirmed somatic driver in Wilms tumour because only one confirmed somatic *FBXW7* point mutation has thus far been reported.^25^ This situation is similar to that of *PIK3CA*. Constitutional mosaic *PIK3CA* mutations predispose to Wilms tumour, whereas somatic mutations at the same residue are common in many cancers but have not been reported in Wilms tumour.^9^

*KDM3B* encodes a histone H3 demethylase that specifically catalyses the demethylation of H3K9Me1 and H3K9Me2 residues, and is required for normal somatic growth in mice.^28^ Tumour-suppressive and tumour-promoting KDM3B activities have been proposed in leukaemia, although somatic driver *KDM3B* mutations have not been reported. Finally, there is very little known about the functions of *NYNRIN*, though NYN domains are thought to be involved in RNA processing and NYNRIN has been implicated in microRNA–mRNA regulation.^29^

The diverse functions of these four new Wilms tumour predisposition genes mirror the broad range of biological processes in which known Wilms tumour predisposition genes operate, as shown by our functional enrichment analysis (appendix). Functional exploration of these genes was beyond the scope of our study, but we hope our results might encourage such assessments, which will probably provide novel insights into oncogenesis and kidney development.

Our analyses were designed to identify tumour suppressor genes in which constitutional truncating mutations predisposed to cancer, but were not designed to identify other mechanisms of cancer predisposition. For example, it is very possible that non-truncating coding variation might be contributing to familial and non-familial Wilms tumour, and future analyses to investigate this would be worthwhile. Non-coding, epigenetic, and mosaic abnormalities are all known to be relevant to Wilms tumour predisposition but were not investigated in our study. Notably, none of the known Wilms tumour predisposition genes are within the regions on chromosomes 2p24, 11q14, 5q14, 22q12, and Xp22 identified in a genome-wide association study^30^ of Wilms tumour, and the causal mechanisms underlying the associations in that study are unknown. Additionally, the mutations at 17q21 responsible for *FWT1*-linked families have not yet been discovered.^2^

Genetic predisposition to Wilms tumour exhibits remarkable heterogeneity, and this is particularly noteworthy because childhood cancers are generally assumed to be aetiologically simpler than adult cancers. Furthermore, our study provides strong evidence that further genetic, genomic, or epigenetic Wilms tumour predisposition factors exist, because only a third of the familial Wilms tumour pedigrees we investigated have been explained. Any further familial Wilms tumour genes discovered will be highly likely to contribute also to non-familial Wilms tumour.

Our study reveals new insights into the complexity, mechanisms, and clinical implications of Wilms tumour predisposition. Although our understanding of the genetic landscape of Wilms tumour predisposition is still far from complete, the available knowledge has considerable scientific and clinical use. Given the extensive heterogeneity and the absence of family history or additional clinical features in many individuals with a mutation, we believe routine genetic testing in all individuals with Wilms tumour would be scientifically and clinically valuable.
