# Supplementary material for: Identification of new Wilms tumour predisposition genes: an exome sequencing study
Source: Lancet Child Adolesc Health. 2019 May;3(5):322–31. doi: 10.1016/S2352-4642(19)30018-5 (PMC6472290; doi:10.1016/S2352-4642(19)30018-5)
Supplement: Supplementary appendix [file mmc1.pdf]

THE LANCET
Child & Adolescent Health

Supplementary appendix

This appendix formed part of the original submission and has been peer reviewed. We post it as supplied by the authors.

Supplement to: Mahamdallie S, Yost S, Poyastro-Pearson E, et al. Identification of new Wilms tumour predisposition genes: an exome sequencing study. *Lancet Child Adolesc Health* 2019; published online March 15. http://dx.doi.org/10.1016/S2352-4642(19)30018-5.

Supplementary Material

Identifying new Wilms tumor predisposition genes - an exome sequencing study

Shazia Mahamdallie *et al*.

Supplementary Figure 1. Mutations and pedigrees of individuals with childhood cancer due to constitutional mutations in *TRIM28*, *FBXW7*, *NYNRIN*, *KDM3B* or *CDC73*.
Supplementary Table 1. Number and types of childhood cancer constitutional exomes analysed.
Supplementary Table 2. Familial Wilms tumor pedigrees analysed.
Supplementary Table 3. Number and types of adult cancer constitutional exomes (TCGA) analysed.
Supplementary Table 4. Top 50 out of 872 significantly enriched pathways involving Wilms tumor predisposition genes.
Supplementary Note. The FACT collaboration

Supplementary Figure 1. Mutations and pedigrees of individuals with childhood cancer due to constitutional mutations in *TRIM28*, *FBXW7*, *NYNRIN*, *KDM3B* or *CDC73*.

TRIM28

ID_0477

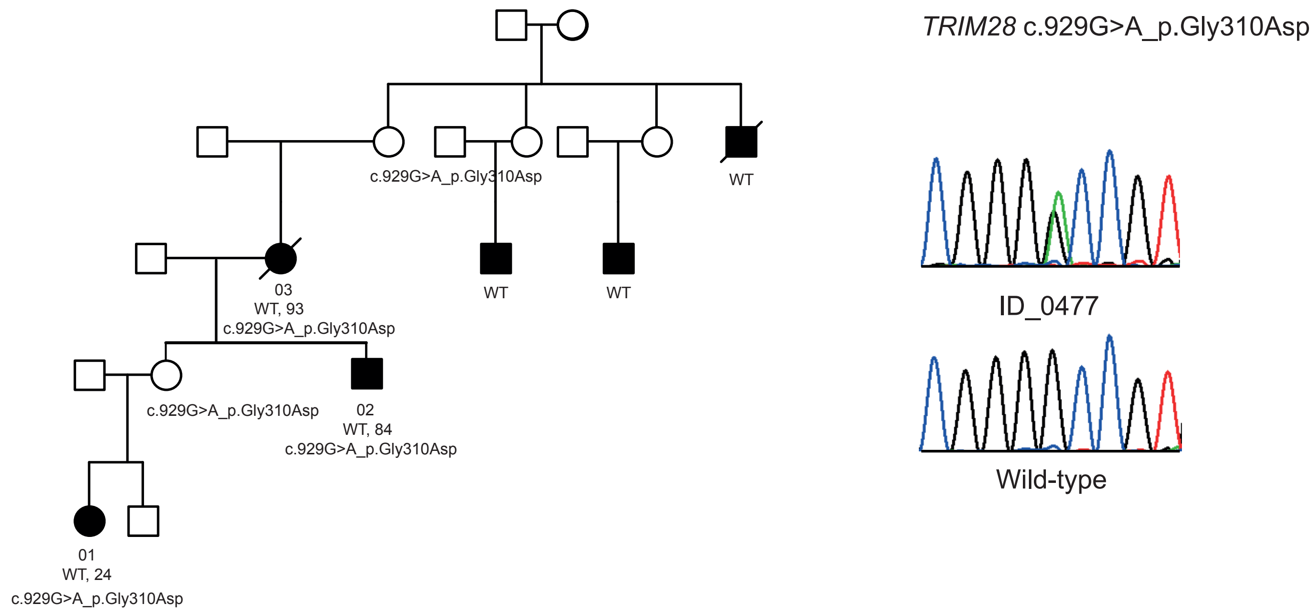

ID_0498

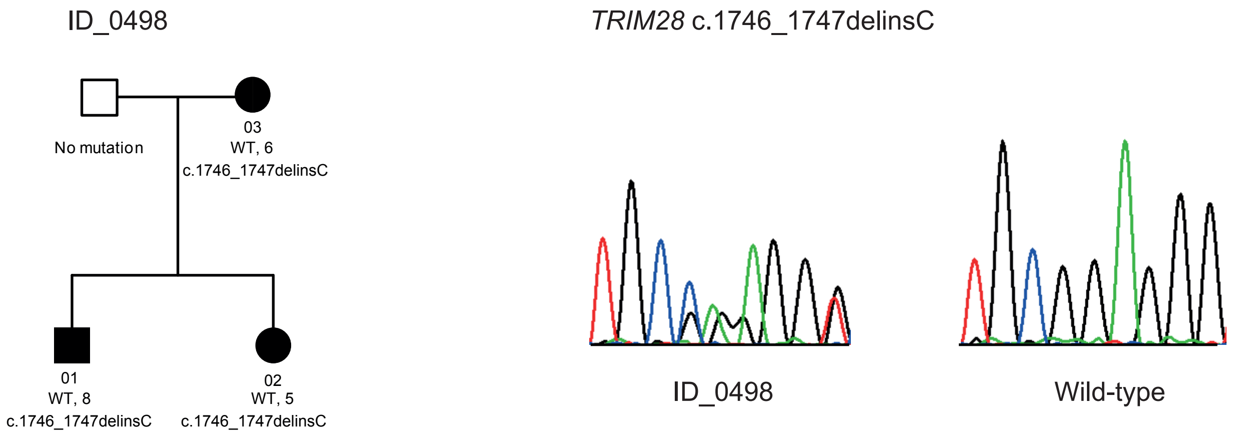

ID_0487

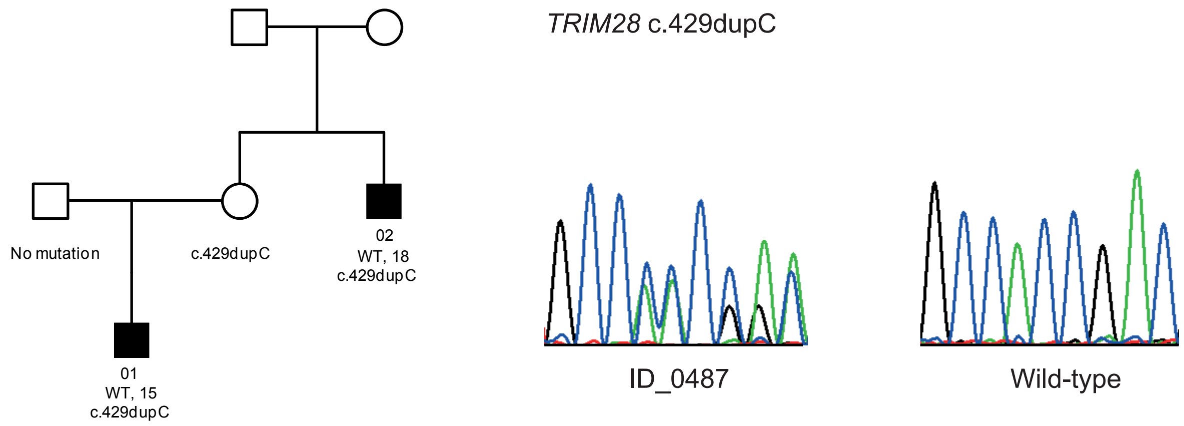

ID_0506

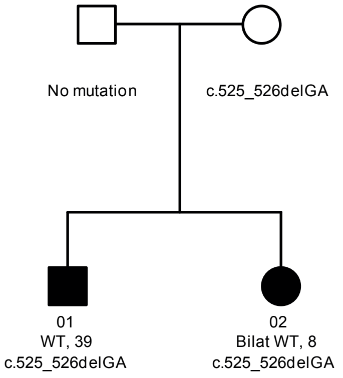

TRIM28 c.525_526delGA

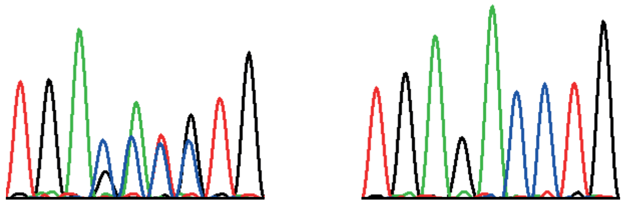

ID_0506 Wild-type

ID_7487

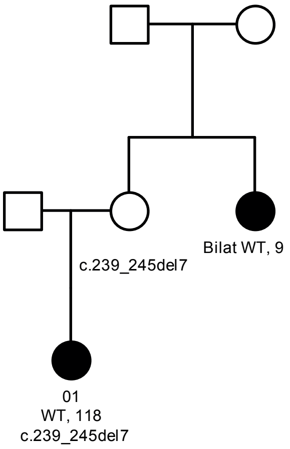

TRIM28 c.239_245del7

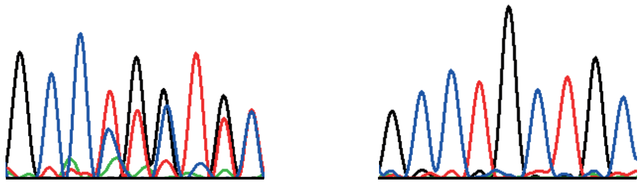

ID_7487 Wild-type

ID_1982

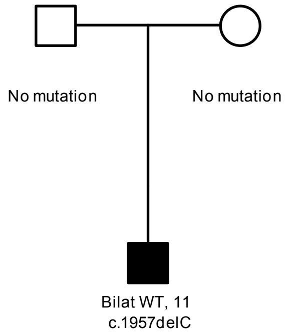

TRIM28 c.1957delC

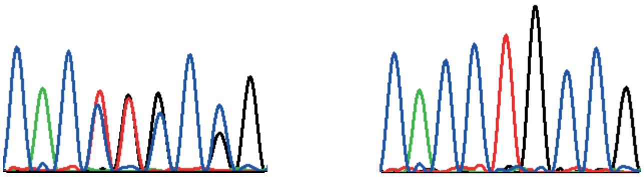

ID_1982 Wild-type

ID_6530

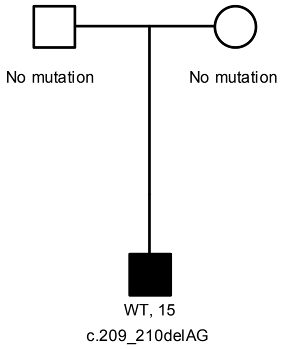

TRIM28 c.209_210delAG

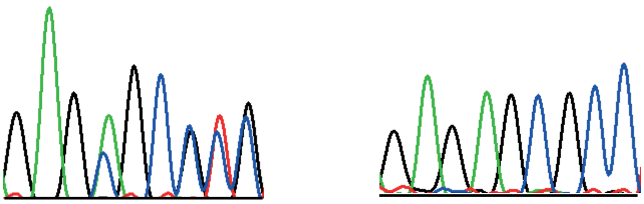

ID_6530 Wild-type

ID_1969 *TRIM28* c.840-2A>G

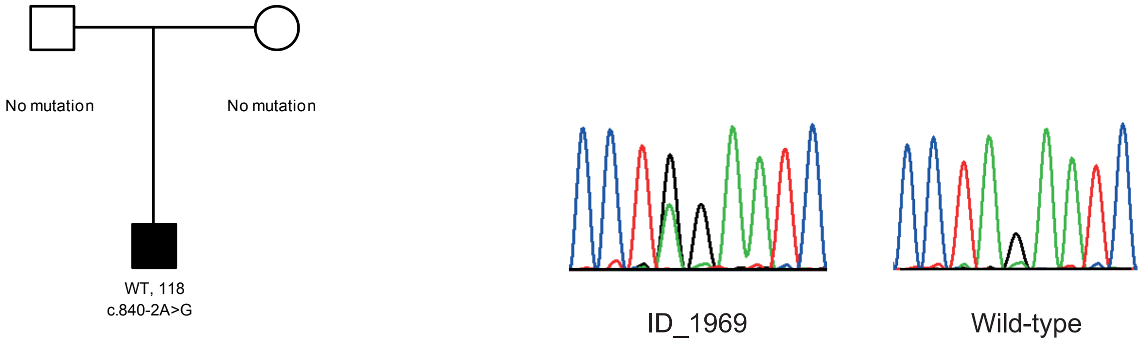

ID_7574 *TRIM28* c.2508A>G_p.X836TrpextX?

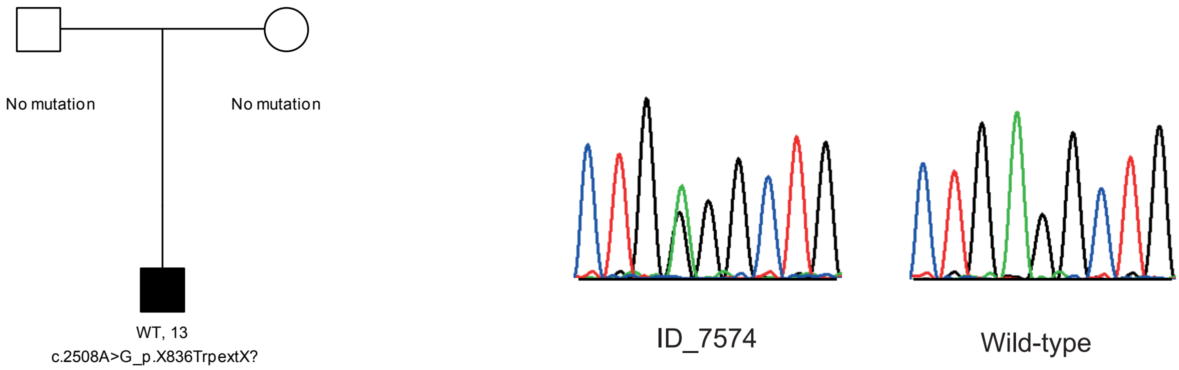

ID_0902 *TRIM28* c.1250C>A_p.Ser417X

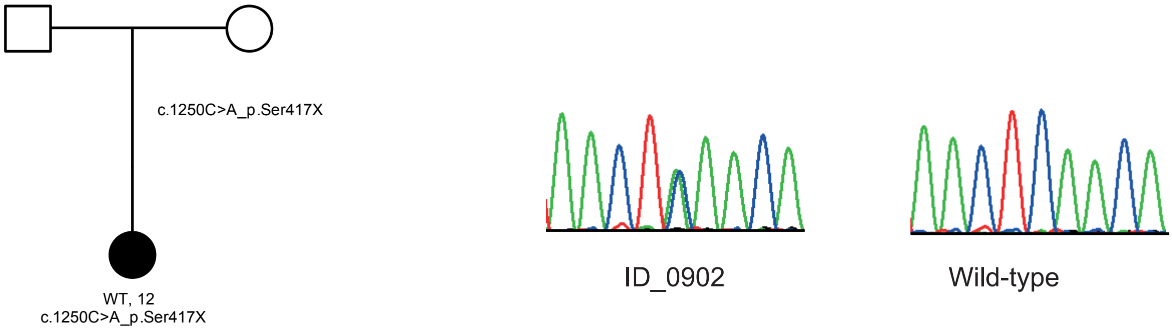

ID_0692 *TRIM28* c.1459C>T_p.Arg487X

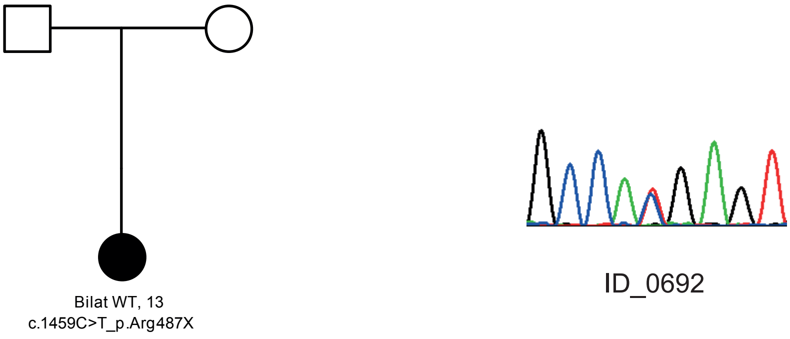

ID_6671

TRIM28 c.688C>T_p.Arg230X

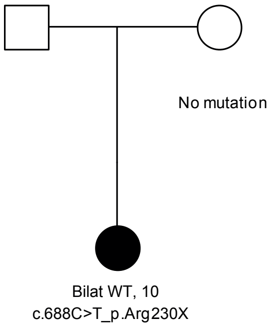

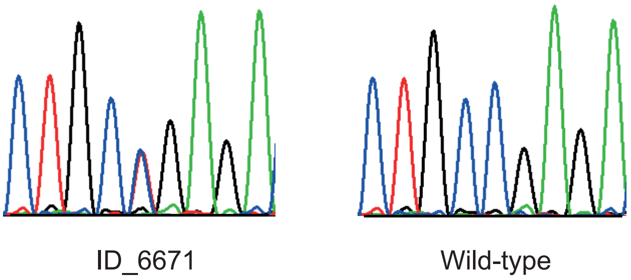

ID_0796

TRIM28 c.1085T>A_p.Leu362X

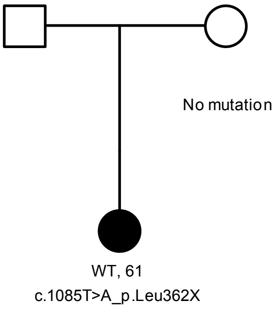

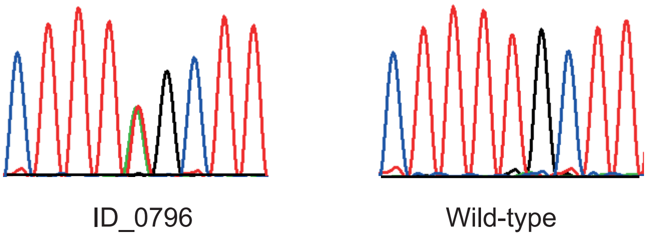

ID_0866

TRIM28 c.1300_1301dupAA

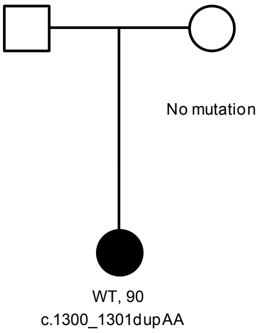

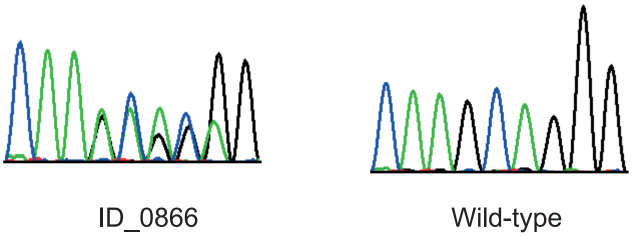

ID_0936

TRIM28 c.1150G>T_p.Glu384X

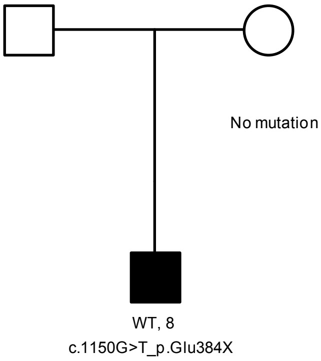

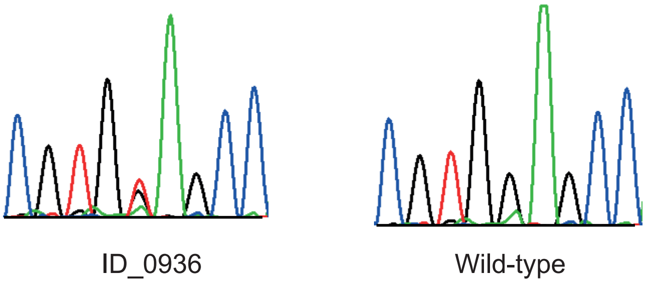

FBXW7

ID_0811

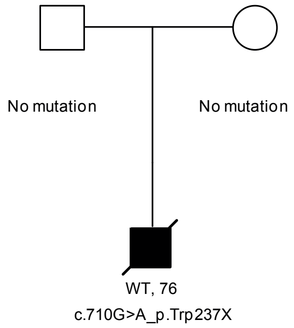

FBXW7 c.710G>A_p.Trp237X

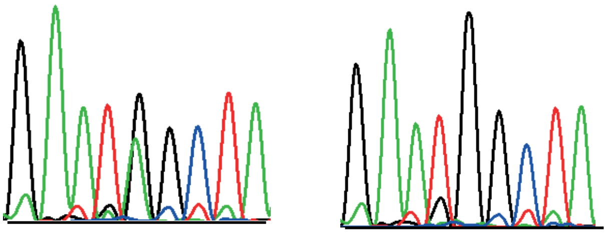

ID_0811 Wild-type

ID_2084

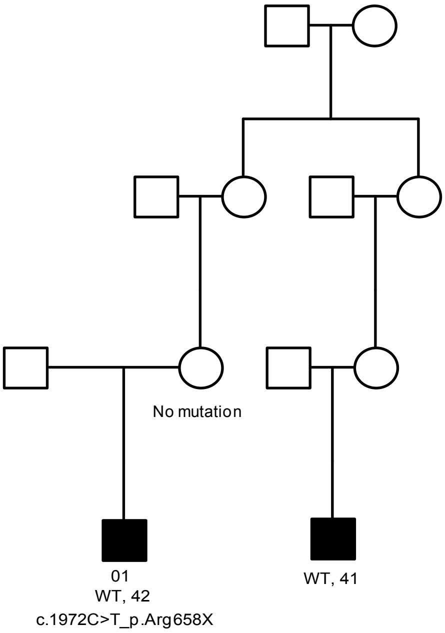

FBXW7 c.1972C>T_p.Arg658X

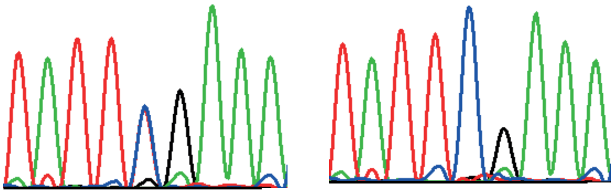

ID_2084 Wild-type

ID_0592

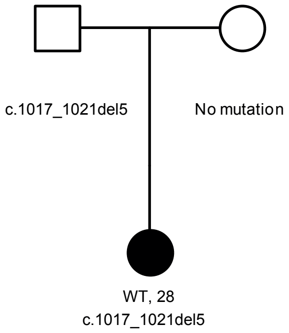

FBXW7 c.1017_1021del5

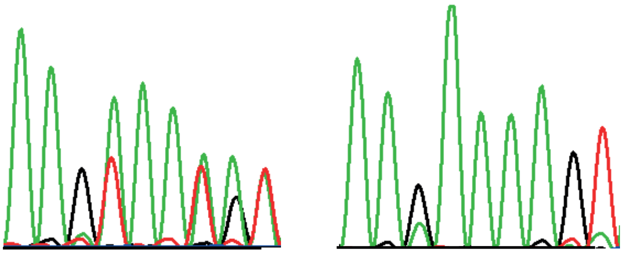

ID_0592 Wild-type

ID_1227

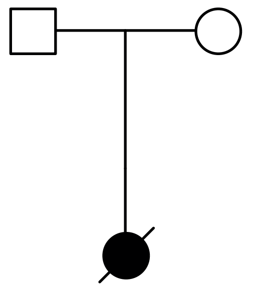

FBXW7 c.670C>T_p.Arg224X

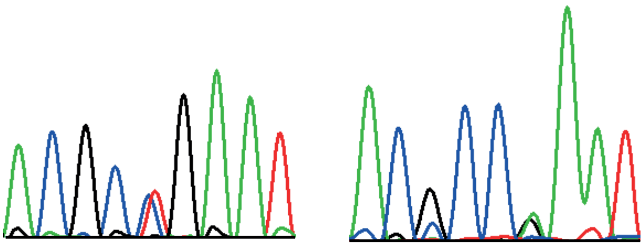

ID_7520

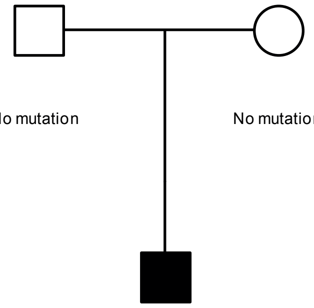

FBXW7 c.1753A>T_p.Ser585Cys

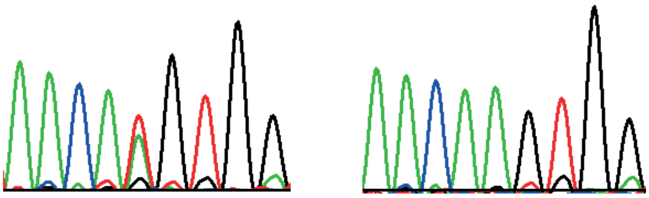

NYNRIN

ID_0493

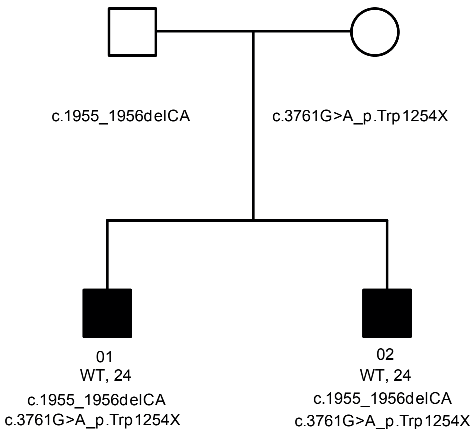

NYNRIN c.1955_1956delCA

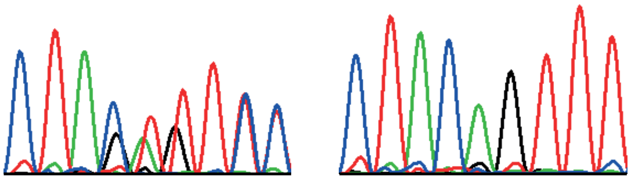

ID_0493 Wild-type

NYNRIN c.3761G>A_p.Trp1254X

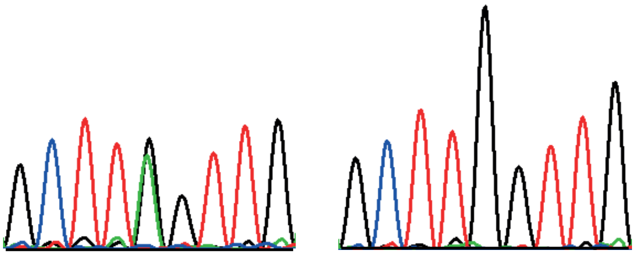

ID_0493 Wild-type

ID_6049

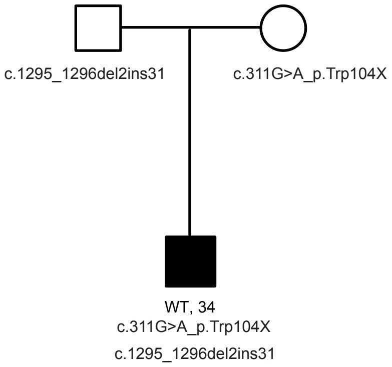

NYNRIN c.311G>A_p.Trp104X

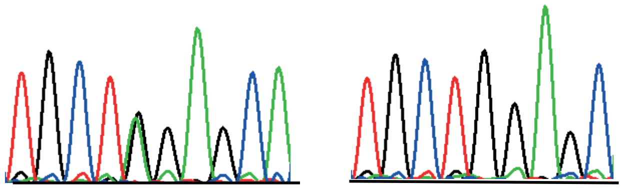

ID_6049 Wild-type

NYNRIN c.1295_1296del2ins31

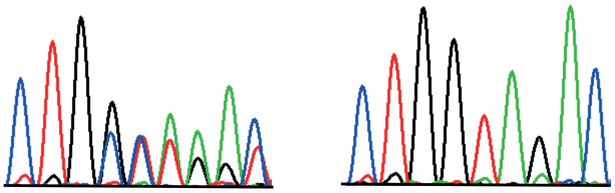

ID_6049 Wild-type

NB the full notation for c.1295_1996del2ins31 is c.1295_1296delinsCCTAGTGCAGAAAGCCCAGCCTAGTGCAGAA

KDM3B

ID_7225

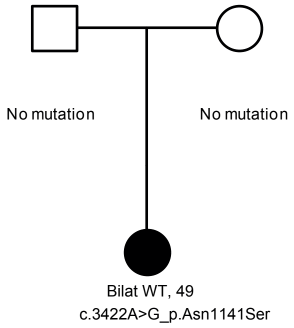

KDM3B c.3422A>G_p.Asn1141Ser

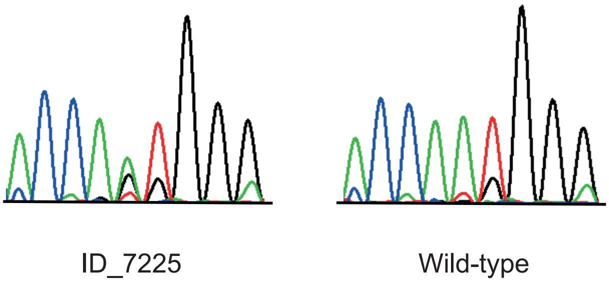

ID_2086

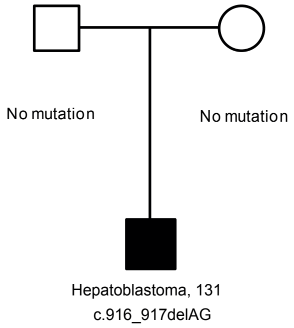

KDM3B c.916_917delAG

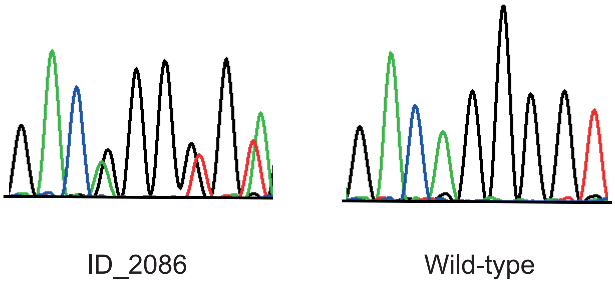

CDC73

ID_6491

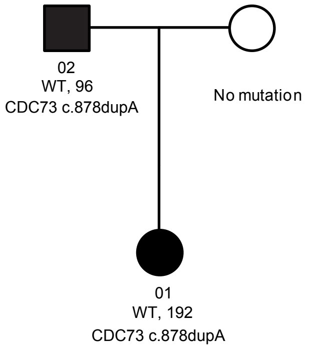

ID	Genomic coordinate	REF	ALT	GT	TR	TC	MRP
ID_6491_01	1:193119482	T	TA	1/0	243	553	44
ID_6491_02	1:193119482	T	TA	1/0	154	342	45

Sanger sequencing chromatograms validating the constitutional mutations identified in the childhood and adult exome data, and any available relatives. The *CDC73* mutation called from exome data was validated using the TruSight Cancer Panel. WT = Wilms tumor. Bilat = Bilateral. ", X" Age of diagnosis given in months. Filled symbols indicates affected with cancer.

Supplementary Table 1. Number and types of childhood cancer constitutional exomes analysed.

Cancer	Number of Samples
Wilms tumor	890
Hepatoblastoma	109
Ependymoma	51
Rhabdoid tumor	40
Clear cell sarcoma of kidney	18
Medulloblastoma	18
Neuroblastoma	17
Choroid plexus tumor	15
Pleuroplumonary blastoma	10
Phaeochromocytoma/paraganglioma	8
Pineoblastoma	6
Embryonal tumor with multilayered rosettes	5
Pancreatoblastoma	5
Embryonal sarcoma of the liver	5
Melanoma	4
Acute lymphoblastic leukaemia	3
Rhabdomyosarcoma	3
Astrocytoma	2
Germ cell tumor	2
Lymphoma	2
Pineal tumor	2
Sarcoma	2
Sex cord-stromal tumor	2
Gonadoblastoma	1
Hepatocellular	1
Neurofibrosarcoma	1
Osteosarcoma	1
Skin cancer (non-melanoma)	1

Supplementary Table 2. Familial Wilms tumour pedigrees analysed.

ID	WT cases in family	WT cases exomed	Cause	Reference (PMID)	Note
ID_0475	9	2		8696342	FWT1-linked, Also called Family 480
ID_0476	2		BRCA2 (biallelic)	15689453	Also called WILMS2
ID_0477	3	3	TRIM28	This study	
ID_0478	2	2		This study	
ID_0479	2		WT1	10905890	Also called WILMS5
ID_0480	2	2		This study	
ID_0481	2		REST	26551668	
ID_0482	2		REST	26551668	
ID_0483	2	2		This study	
ID_0484	2		CTR9	25099282	
ID_0485	2	2		This study	
ID_0486	3	3		This study	
ID_0487	2	2	TRIM28	This study	
ID_0488	2		CTR9	25099282	
ID_0489	2	2		This study	
ID_0490	2	2		This study	
ID_0491	2	1		This study	
ID_0492	2	1		This study	
ID_0493	2	2	NYNRIN	This study	
ID_0494	4	4		This study	
ID_0495	2	2		This study	
ID_0496	3		H19 hypermethylation	18836444	Also called Family 22
ID_0497	2			None	H19 hypermethylation in one case, does not segregate
ID_0498	3	3	TRIM28	This study	
ID_0499	2	2		This study	
ID_0500	2	1		This study	
ID_0501	2	2		This study	
ID_0502	2	1		This study	
ID_0503	2		WT1	None	
ID_0504	2	2		This study	
ID_0505	4	1		9860296	FWT1-linked, Also called K1104
ID_0506	2	1	TRIM28	This study	
ID_0507	2	2		This study	
ID_0508	2	2		This study	
ID_0509	2		REST	26551668	
ID_0510	2	2		This study	
ID_0349	2	2		This study	
ID_0462	2	1		This study	
ID_0351	2		H19 hypermethylation	None	
ID_0320	2	1		This study	
ID_0689	2		REST	26551668	
ID_2003	2		WT1	None	
ID_2006	3	3		This study	
ID_2097	2	2		This study	
ID_1131	2	2		This study	
ID_3679	2	2		This study	
ID_2084	2	1		This study	FBXW7 in one case, does not segregate
ID_3727	2		CTR9	25099282	
ID_5265	2	2		This study	
ID_5673	2	2		This study	
ID_5737	2	2		This study	
ID_5804	2	2		This study	
ID_1203	2		REST	None	
ID_6138	2	2		This study	
ID_6150	2	1		This study	
ID_6261	2	2		This study	
ID_6189	2	2		This study	
ID_6698	2	2		This study	
ID_6491	2	2	CDC73	This study	
ID_7208	3	1		This study	
ID_6991	2	1		This study	
ID_7309	2		WT1	None	
ID_7455	2	2		This study	
ID_7487	2	1	TRIM28	This study	
ID_7648	2	2		This study	

Supplementary Table 3. Number and types of adult cancer constitutional exomes (TCGA) analysed.

Cancer	Full Description	Number of Samples
BR	Breast invasive carcinoma	779
UCEC	Uterine Corpus Endometrioid Carcinoma	488
THCA	Thyroid carcinoma	480
LUAD	Lung adenocarcinoma	470
HNSC	Head and Neck squamous cell carcinoma	452
LGG	Brain Lower Grade Glioma	430
LUSC	Lung squamous cell carcinoma	422
SKCM	Skin Cutaneous Melanoma	402
GBM	Glioblastoma multiforme	381
OV	Ovarian serous cystadenocarcinoma	370
KIRC	Kidney renal clear cell carcinoma	363
STAD	Stomach adenocarcinoma	347
PRAD	Prostate adenocarcinoma	345
COAD	Colon adenocarcinoma	264
CESC	Cervical squamous cell carcinoma and endocervical adenocarcinoma	238
BLCA	Bladder Urothelial Carcinoma	230
LIHC	Liver hepatocellular carcinoma	191
KIRP	Kidney renal papillary cell carcinoma	190
ESCA	Esophageal carcinoma	141
SARC	Sarcoma	118
PAAD	Pancreatic adenocarcinoma	107
READ	Rectum adenocarcinoma	106
ACC	Adrenocortical carcinoma	80
KICH	Kidney Chromophobe	66
PCPG	Pheochromocytoma and Paraganglioma	60
UCS	Uterine Carcinosarcoma	57
DLBC	Lymphoid Neoplasm Diffuse Large B-cell Lymphoma	33
LAML	Acute Myeloid Leukemia	22

Supplementary Table 4. Top 50 out of 872 significantly enriched pathways involving Wilms tumor predisposition genes.

p-value	# of Pathway genes	# of WT genes	# of WT genes in Pathway	Pathway ID	Pathway name	Pathway depth	WT genes in Pathway
3.59E-11	1167	21	15	GO:0051276	chromosome organization	4	TRIP13,REST,TRIM37,FBXW7,KDM3B,TRIM28,CDC73,BRCA2,TP53,DIS3L2,BUB1B,IGF2,ASXL1,BLM,CTR9
1.62E-08	463	21	10	GO:0016569	covalent chromatin modification	5	REST,TRIM37,KDM3B,TRIM28,CDC73,BRCA2,TP53,IGF2,ASXL1,CTR9
5.47E-08	5299	21	20	GO:0090304	nucleic acid metabolic process	5	TRIP13,PALB2,REST,DICER1,TRIM37,FBXW7,KDM3B,PIK3CA,TRIM28,CDC73,BRCA2,TP53,DIS3L2,GPC3,IGF2,ASXL1,WT1,BLM,CTR9,NYNRIN
9.24E-08	4626	21	19	GO:0048523	negative regulation of cellular process	3	TRIP13,PALB2,REST,DICER1,TRIM37,FBXW7,PIK3CA,TRIM28,CDC73,BRCA2,TP53,DIS3L2,GPC3,BUB1B,IGF2,ASXL1,WT1,BLM,CTR9
0.000000176	450	21	9	GO:0016570	histone modification	6	REST,TRIM37,KDM3B,CDC73,BRCA2,TP53,IGF2,ASXL1,CTR9
0.000000255	5937	21	20	GO:0006139	nucleobase-containing compound metabolic process	4	TRIP13,PALB2,REST,DICER1,TRIM37,FBXW7,KDM3B,PIK3CA,TRIM28,CDC73,BRCA2,TP53,DIS3L2,GPC3,IGF2,ASXL1,WT1,BLM,CTR9,NYNRIN
0.000000365	6096	21	20	GO:0046483	heterocycle metabolic process	3	TRIP13,PALB2,REST,DICER1,TRIM37,FBXW7,KDM3B,PIK3CA,TRIM28,CDC73,BRCA2,TP53,DIS3L2,GPC3,IGF2,ASXL1,WT1,BLM,CTR9,NYNRIN
0.000000365	6137	21	20	GO:0006725	cellular aromatic compound metabolic process	3	TRIP13,PALB2,REST,DICER1,TRIM37,FBXW7,KDM3B,PIK3CA,TRIM28,CDC73,BRCA2,TP53,DIS3L2,GPC3,IGF2,ASXL1,WT1,BLM,CTR9,NYNRIN
0.000000365	5209	21	19	GO:0048519	negative regulation of biological process	2	TRIP13,PALB2,REST,DICER1,TRIM37,FBXW7,PIK3CA,TRIM28,CDC73,BRCA2,TP53,DIS3L2,GPC3,BUB1B,IGF2,ASXL1,WT1,BLM,CTR9
0.000000501	777	21	10	GO:0006325	chromatin organization	3	REST,TRIM37,KDM3B,TRIM28,CDC73,BRCA2,TP53,IGF2,ASXL1,CTR9
0.000000501	6338	21	20	GO:1901360	organic cyclic compound metabolic process	3	TRIP13,PALB2,REST,DICER1,TRIM37,FBXW7,KDM3B,PIK3CA,TRIM28,CDC73,BRCA2,TP53,DIS3L2,GPC3,IGF2,ASXL1,WT1,BLM,CTR9,NYNRIN
0.00000057	1408	21	12	GO:2000113	negative regulation of cellular macromolecule biosynthetic process	6	REST,DICER1,TRIM37,FBXW7,TRIM28,CDC73,BRCA2,TP53,IGF2,WT1,BLM,CTR9
0.000000594	1423	21	12	GO:0045934	negative regulation of nucleobase-containing compound metabolic process	5	REST,DICER1,TRIM37,FBXW7,TRIM28,CDC73,BRCA2,TP53,IGF2,WT1,BLM,CTR9
0.000000869	1481	21	12	GO:0010558	negative regulation of macromolecule biosynthetic process	5	REST,DICER1,TRIM37,FBXW7,TRIM28,CDC73,BRCA2,TP53,IGF2,WT1,BLM,CTR9
0.000000898	6634	21	20	GO:0034641	cellular nitrogen compound metabolic process	3	TRIP13,PALB2,REST,DICER1,TRIM37,FBXW7,KDM3B,PIK3CA,TRIM28,CDC73,BRCA2,TP53,DIS3L2,GPC3,IGF2,ASXL1,WT1,BLM,CTR9,NYNRIN
0.00000117	1538	21	12	GO:0031327	negative regulation of cellular biosynthetic process	5	REST,DICER1,TRIM37,FBXW7,TRIM28,CDC73,BRCA2,TP53,IGF2,WT1,BLM,CTR9
0.00000128	1559	21	12	GO:0009890	negative regulation of biosynthetic process	4	REST,DICER1,TRIM37,FBXW7,TRIM28,CDC73,BRCA2,TP53,IGF2,WT1,BLM,CTR9
0.00000139	25	21	4	GO:0071514	genetic imprinting	4	PIK3CA,TRIM28,IGF2,CTR9
0.00000139	2977	21	15	GO:0009892	negative regulation of metabolic process	3	REST,DICER1,TRIM37,FBXW7,PIK3CA,TRIM28,CDC73,BRCA2,TP53,DIS3L2,GPC3,IGF2,WT1,BLM,CTR9
0.00000139	4210	21	17	GO:0019219	regulation of nucleobase-containing compound metabolic process	4	TRIP13,REST,DICER1,TRIM37,FBXW7,KDM3B,PIK3CA,TRIM28,CDC73,BRCA2,TP53,GPC3,IGF2,ASXL1,WT1,BLM,CTR9
0.00000174	8350	21	21	GO:0044260	cellular macromolecule metabolic process	3	TRIP13,PALB2,REST,DICER1,TRIM37,FBXW7,KDM3B,PIK3CA,TRIM28,CDC73,BRCA2,TP53,DIS3L2,GPC3,BUB1B,IGF2,ASXL1,WT1,BLM,CTR9,NYNRIN
0.00000174	975	21	10	GO:0006259	DNA metabolic process	6	TRIP13,PALB2,DICER1,FBXW7,PIK3CA,TRIM28,BRCA2,TP53,BLM,NYNRIN
0.00000174	964	21	10	GO:0040007	growth	1	PALB2,PIK3CA,TRIM28,CDC73,BRCA2,TP53,GPC3,IGF2,WT1,CTR9
0.00000174	706	21	9	GO:0010564	regulation of cell cycle process	4	TRIP13,TRIM37,FBXW7,CDC73,BRCA2,TP53,BUB1B,IGF2,BLM
0.00000174	303	21	7	GO:0010948	negative regulation of cell cycle process	5	TRIP13,TRIM37,FBXW7,CDC73,TP53,BUB1B,BLM
0.00000174	2537	21	14	GO:0031324	negative regulation of cellular metabolic process	4	REST,DICER1,TRIM37,FBXW7,PIK3CA,TRIM28,CDC73,BRCA2,TP53,GPC3,IGF2,WT1,BLM,CTR9
0.00000206	3751	21	16	GO:2001141	regulation of RNA biosynthetic process	6	TRIP13,REST,DICER1,TRIM37,FBXW7,KDM3B,TRIM28,CDC73,BRCA2,TP53,GPC3,IGF2,ASXL1,WT1,BLM,CTR9
0.00000206	3743	21	16	GO:1903506	regulation of nucleic acid-templated transcription	7	TRIP13,REST,DICER1,TRIM37,FBXW7,KDM3B,TRIM28,CDC73,BRCA2,TP53,GPC3,IGF2,ASXL1,WT1,BLM,CTR9
0.00000215	6120	21	19	GO:0016043	cellular component organization	2	TRIP13,REST,DICER1,TRIM37,FBXW7,KDM3B,PIK3CA,TRIM28,CDC73,BRCA2,TP53,DIS3L2,GPC3,BUB1B,IGF2,ASXL1,WT1,BLM,CTR9
0.00000245	747	21	9	GO:0008285	negative regulation of cell proliferation	4	REST,DICER1,FBXW7,CDC73,BRCA2,TP53,DIS3L2,GPC3,WT1
0.00000299	3872	21	16	GO:0006351	transcription, DNA-templated	8	TRIP13,REST,DICER1,TRIM37,FBXW7,KDM3B,TRIM28,CDC73,BRCA2,TP53,GPC3,IGF2,ASXL1,WT1,BLM,CTR9
0.00000309	3889	21	16	GO:0097659	nucleic acid-templated transcription	7	TRIP13,REST,DICER1,TRIM37,FBXW7,KDM3B,TRIM28,CDC73,BRCA2,TP53,GPC3,IGF2,ASXL1,WT1,BLM,CTR9
0.0000031	6297	21	19	GO:0071840	cellular component organization or biogenesis	1	TRIP13,REST,DICER1,TRIM37,FBXW7,KDM3B,PIK3CA,TRIM28,CDC73,BRCA2,TP53,DIS3L2,GPC3,BUB1B,IGF2,ASXL1,WT1,BLM,CTR9
0.0000031	3905	21	16	GO:0032774	RNA biosynthetic process	6	TRIP13,REST,DICER1,TRIM37,FBXW7,KDM3B,TRIM28,CDC73,BRCA2,TP53,GPC3,IGF2,ASXL1,WT1,BLM,CTR9
0.00000361	2779	21	14	GO:0010605	negative regulation of macromolecule metabolic process	4	REST,DICER1,TRIM37,FBXW7,TRIM28,CDC73,BRCA2,TP53,DIS3L2,GPC3,IGF2,WT1,BLM,CTR9
0.00000361	3955	21	16	GO:0051252	regulation of RNA metabolic process	5	TRIP13,REST,DICER1,TRIM37,FBXW7,KDM3B,TRIM28,CDC73,BRCA2,TP53,GPC3,IGF2,ASXL1,WT1,BLM,CTR9
0.00000361	798	21	9	GO:0000122	negative regulation of transcription by RNA polymerase II	9	REST,DICER1,TRIM37,TRIM28,CDC73,TP53,IGF2,WT1,CTR9
0.00000364	1443	21	11	GO:0045893	positive regulation of transcription, DNA-templated	8	REST,FBXW7,TRIM28,CDC73,BRCA2,TP53,IGF2,ASXL1,WT1,BLM,CTR9
0.00000506	4789	21	17	GO:0016070	RNA metabolic process	6	TRIP13,REST,DICER1,TRIM37,FBXW7,KDM3B,TRIM28,CDC73,BRCA2,TP53,DIS3L2,GPC3,IGF2,ASXL1,WT1,BLM,CTR9
0.00000552	4823	21	17	GO:0010468	regulation of gene expression	4	REST,DICER1,TRIM37,FBXW7,KDM3B,PIK3CA,TRIM28,CDC73,BRCA2,TP53,DIS3L2,GPC3,IGF2,ASXL1,WT1,BLM,CTR9
0.00000565	2384	21	13	GO:0051172	negative regulation of nitrogen compound metabolic process	4	REST,DICER1,TRIM37,FBXW7,TRIM28,CDC73,BRCA2,TP53,GPC3,IGF2,WT1,BLM,CTR9
0.0000058	1528	21	11	GO:1902680	positive regulation of RNA biosynthetic process	6	REST,FBXW7,TRIM28,CDC73,BRCA2,TP53,IGF2,ASXL1,WT1,BLM,CTR9
0.0000058	1527	21	11	GO:1903508	positive regulation of nucleic acid-templated transcription	7	REST,FBXW7,TRIM28,CDC73,BRCA2,TP53,IGF2,ASXL1,WT1,BLM,CTR9
0.00000642	5716	21	18	GO:0010467	gene expression	4	TRIP13,REST,DICER1,TRIM37,FBXW7,KDM3B,PIK3CA,TRIM28,CDC73,BRCA2,TP53,DIS3L2,GPC3,IGF2,ASXL1,WT1,BLM,CTR9
0.00000837	4256	21	16	GO:0010556	regulation of macromolecule biosynthetic process	4	TRIP13,REST,DICER1,TRIM37,FBXW7,KDM3B,TRIM28,CDC73,BRCA2,TP53,GPC3,IGF2,ASXL1,WT1,BLM,CTR9
0.00000875	1601	21	11	GO:0051254	positive regulation of RNA metabolic process	6	REST,FBXW7,TRIM28,CDC73,BRCA2,TP53,IGF2,ASXL1,WT1,BLM,CTR9
0.00000982	1249	21	10	GO:0022402	cell cycle process	3	TRIP13,TRIM37,FBXW7,CDC73,BRCA2,TP53,DIS3L2,BUB1B,IGF2,BLM
0.0000116	3710	21	15	GO:0006996	organelle organization	3	TRIP13,REST,TRIM37,FBXW7,KDM3B,TRIM28,CDC73,BRCA2,TP53,DIS3L2,BUB1B,IGF2,ASXL1,BLM,CTR9
0.0000116	4382	21	16	GO:0031326	regulation of cellular biosynthetic process	4	TRIP13,REST,DICER1,TRIM37,FBXW7,KDM3B,TRIM28,CDC73,BRCA2,TP53,GPC3,IGF2,ASXL1,WT1,BLM,CTR9

Supplementary Note. The FACT collaboration

The Factors Associated with Childhood Tumors (FACT) collaboration undertakes recruitment of families and collection of samples from individuals with childhood cancer. It includes the following collaborators:

A Abudu, J Anderson, G Anyaegbu, R Beckingham, J Birch, H Bishop, L Bishton, E Blanco, B Brennan, M Bridgens, R Browning, C Brunton, A Burke, Q Campbell Hewson, M Capra, S Carter, B Castle, C Cawley, E Charnock, J Chisholm, T Chowdhury, R Collins, J Cooper, R Cox, S Crabtree, T Crowe, F Davies, A Edgar, R Eeles, M Elliott, L Ellison, J Evans, G Forster, W Foulkes, C Gardiner, A Glaser, J Gray, R Grimer, R Grundy, R Guermech, J Hale, G Halliday, D Hargrave, H Hatcher, J Hayden, S Hemsworth, M Hewitt, K Hilario, E Hincks, D Hobin, M Hoegh-Critchfield, K Howe, L H, well, L Howell, Y Hussain, L Izatt, S Garbutt, A Jenkins, L Jeys, K Johnson, P Kearns, J Kingston, J Kohler, D Lancaster, S Lane, G Levitt, I Lewis, N Lloyd, S Lowis, A Maaz, M Madi, G Makin, C Mallucci, M Manalo, C Mathews, R McAndrew, K McBride, C McConville, H McDowell, S McLoughlin, B Messahel, A Michalski, C Mitchell, J Moreton, B Morland, C Morley-Jacob, P Morrison, J Muller, V Murday, F Mussai, A Ng, J Nicholson, G Nicolin, R O'Keeffe, A O'Meara, JP Kilday, L Palmer, M Parry, A Pearson, A Penn, R Phillips, S Picton, B Pizer, J Powell, L Price, K Pritchard-Jones, M Radford, N Rahman, R Ramanujachar, H Rees, M Ronghe, D Rose, E Ross, A Ryan, T Segrott , SJ Sharman, R Shaw, L Side, O Slater, A Spence, M Stevens, M Taj, K Tatton-Brown, R Tillman, H Traunecker, J Tunnacliffe, C Turner, S Vaidya, J Visser, A Wahlberg, D Walker, K Wheeler, E Whiles, S Wilkins, D Williams, S Wilne, S Wilson, M Wimalendra, S Wool, D Yeomanson and S Zacharoulis.
